# In vivo detection of bile duct pre-cancer with endoscopic light scattering spectroscopy

**DOI:** 10.1038/s41467-022-35780-7

**Published:** 2023-01-07

**Authors:** Douglas K. Pleskow, Mandeep S. Sawhney, Paul K. Upputuri, Tyler M. Berzin, Mark F. Coughlan, Umar Khan, Maria Glyavina, Xuejun Zhang, Liming Chen, Conor J. Sheil, Jonah M. Cohen, Edward Vitkin, Yuri N. Zakharov, Irving Itzkan, Lei Zhang, Le Qiu, Lev T. Perelman

**Affiliations:** 1 Center for Advanced Biomedical Imaging and Photonics, Division of Gastroenterology, Department of Medicine, Beth Israel Deaconess Medical Center, Harvard University, Boston, MA USA; 2 Center for Advanced Endoscopy, Division of Gastroenterology, Department of Medicine, Beth Israel Deaconess Medical Center, Harvard University, Boston, MA USA; 3 Biological and Biomedical Sciences Program, Harvard University, Boston, MA USA

**Keywords:** Bile duct cancer, Optical spectroscopy

## Abstract

Bile duct cancer is the second most common primary liver cancer, with most diagnoses occurring in the advanced stages. This leads to a poor survival rate, which means a technique capable of reliably detecting pre-cancer in the bile duct is urgently required. Unfortunately, radiological imaging lacks adequate accuracy for distinguishing dysplastic and benign biliary ducts, while endoscopic techniques, which can directly assess the bile duct lining, often suffer from insufficient sampling. Here, we report an endoscopic optical light scattering technique for clinical evaluation of the malignant potential of the bile duct. This technique employs an ultraminiature spatial gating fiber optic probe compatible with cholangioscopes and endoscopic retrograde cholangiopancreatography (ERCP) catheters. The probe allowed us to investigate the internal cellular composition of the bile duct epithelium with light scattering spectroscopy (LSS) and phenotypic properties of the underlying connective tissue with diffuse reflectance spectroscopy (DRS). In a pilot in vivo double-blind prospective study involving 29 patients undergoing routine ERCP procedures, the technique detected malignant transformation with 97% accuracy, showing that biliary duct pre-cancer can be reliably identified in vivo non-invasively.

## Abstract (web summary)

Diagnosis of bile duct cancer often occur in advanced stages, leading to poor survival. Here, the authors combine light scattering and diffuse reflectance spectroscopies in a minimally invasive endoscopic technique for directly assessing the malignant potential of the bile duct lining, and demonstrate 97% detection accuracy.

## Introduction

Bile duct cancer, also known as cholangiocarcinoma, is characteristically diagnosed late as most people have no identifiable risk factors^1,2^ for this cancer, resulting in poor outcomes^3^. The only possible treatment options for early-stage cholangiocarcinoma are liver transplantation or surgery before cancer cells spread outside the liver. Even when surgery is successful, the median survival period is ~2 years, depending on the extent of the disease^4^. Because of the poor prognosis, a technique capable of reliably detecting pre-cancer of the bile duct is urgently needed^5^.

Early detection of cholangiocarcinoma is challenging not only because of its asymptomatic character and anatomically hard-to-access location, but also due to low specificity of most diagnostic modalities^6^. Radiological imaging methods, such as ultrasound (US), X-ray computed tomography (CT), magnetic resonance cholangiopancreatography (MRCP), or positron emission tomography–computed tomography (PET-CT) are currently used for the detection of cholangiocarcinoma. However, these imaging modalities are known to suffer from low specificity and accuracy^7^.

Unlike radiological imaging, endoscopic techniques allow direct tissue investigation. They can detect structural changes in multiple top layers of the bile duct during early tumor development, thereby improving sensitivity. For example, endoscopic retrograde cholangiopancreatography (ERCP) can obtain samples of the bile duct lining by brush cytology. While conventional ERCP procedures use X-rays for navigation, recently it became possible to directly visualize the lining of the bile duct in cholangioscopy procedures using ancillary endoscopes, with members of our team pioneering cholangioscopy using the SpyGlass (Boston Scientific) cholangioscope^8^. Cholangioscopy procedures allow collection of the small forceps biopsy specimens, somewhat improving the accuracy of the ERCP diagnosis^9^. However, due to the insufficient sampling, the inherent limitations of both brush cytology and small forceps biopsy limit the sensitivity of ERCP.

Conventional visualization of the bile duct lining has been enhanced by employing narrow-band imaging (NBI)^10^. This technique uses light in the hemoglobin absorption bands of 415 nm and 540 nm, improving the visualization of the mucosa, and probe-based confocal laser endomicroscopy (pCLE)^11^. Studies with NBI demonstrated that NBI improved the identification rate of cancer lesions, as compared with the conventional visualization. However, the detection rate of the pre-cancerous state called dysplasia was not improved^10,12^. Studies with pCLE performed in Germany^13^, France^14^ and by the members of our team^15^, demonstrated that pCLE allows microscopic observation of the bile duct lining, assisting with certain clinical decisions. Despite these advantages, the detection of biliary dysplasia with pCLE was not demonstrated, as the interpretation of the microscopic images obtained with pCLE is not always straightforward.

Another optical medical technology tested for detection of biliary dysplasia is optical coherence tomography (OCT)^16,17^. OCT typically employs low-coherence near-infrared light to visualize the cross-sectional organization of mucosa and is capable of combining high resolution imaging with an ultracompact design needed to access small luminal organs^18^. Both ex vivo^19^ and in vivo^20,21^ OCT studies have demonstrated significant improvements to bile duct cancer detection rates, but detection of dysplasia has not been demonstrated yet. Here, as with pCLE modality, the interpretation of the OCT images requires considerable training.

Fiber-optic label-free light scattering and absorption based optical spectroscopic approaches, such as biomedical diffuse reflectance spectroscopy (DRS)^22–25^, elastic scattering spectroscopy (ESS)^26,27^, or light scattering spectroscopy (LSS)^28–30^, have important advantages for in vivo use in the highly constrained spaces of the bile duct. Not only are they operator independent, but they can also easily be made compatible with even the smallest catheters, require no exogenous markers, and allow rapid investigation of large areas of tissue epithelial lining, detecting both structural and biochemical changes. For example, LSS can probe the cellular organization of the epithelial layer of the digestive tract, detecting the presence of dysplasia and pre-invasive cancer in the esophagus and pancreas^31–33^.

**Fig. 1:**
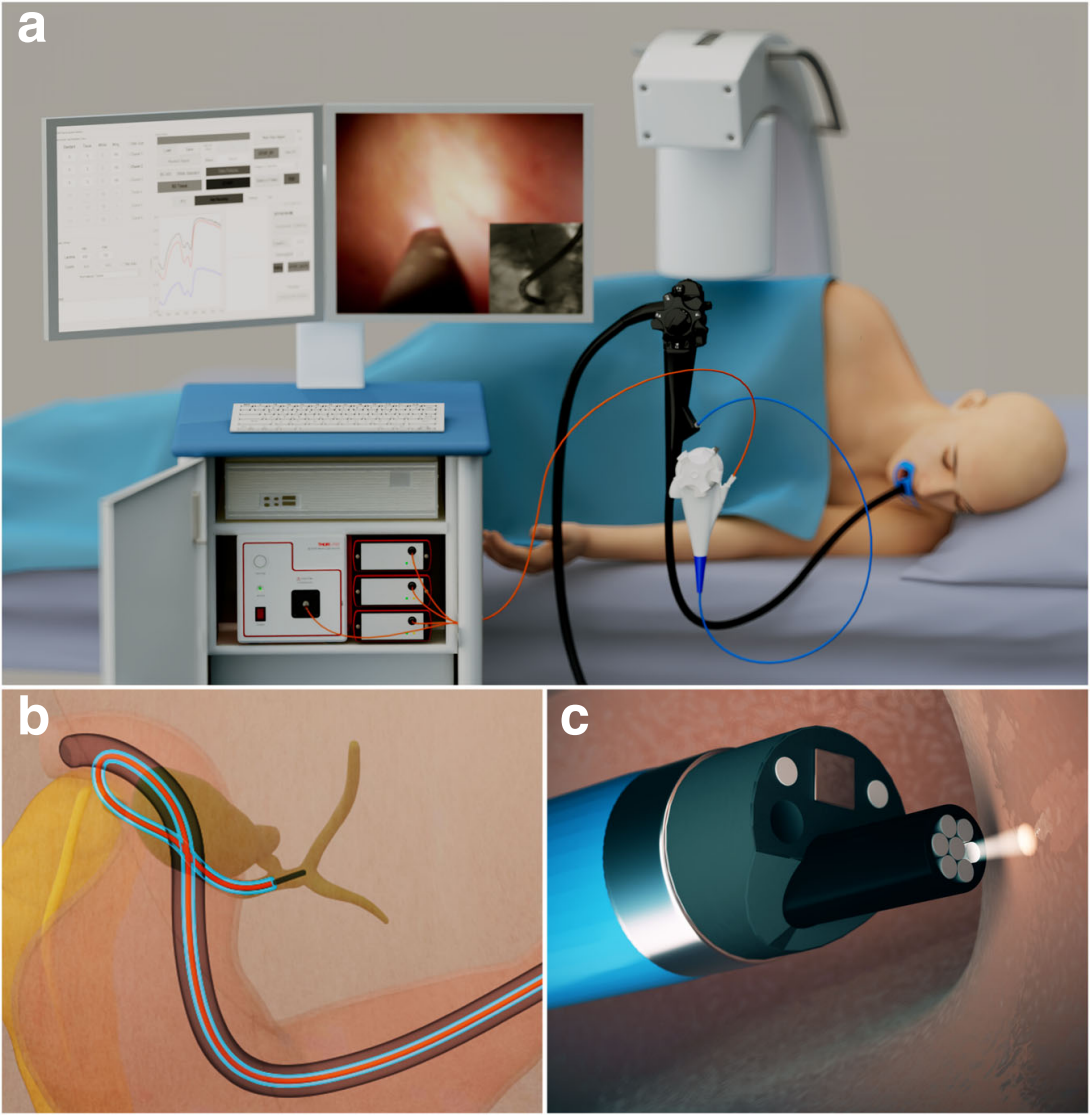
In vivo clinical spectroscopic measurements in the bile duct during endoscopic retrograde cholangiopancreatography (ERCP) procedures. **a** An illustration depicting LSS-DRS measurements of the bile duct wall with the endoscopic light scattering spectroscopy system. A patient is positioned on an X-ray table and a duodenoscope with a cholangioscope placed inside the duodenoscope working channel is inserted into the duodenum. The miniature LSS-DRS fiber optic probe with the delivery fiber coupled to a broadband xenon light source and collection fibers coupled to three spectrometers is inserted into the working channel of a cholangioscope. **b** The LSS-DRS fiber optic probe is passed into the pancreaticobiliary system. **c** The probe illuminates a location of the internal bile duct epithelial lining and the spectra are acquired automatically when the probe is in close proximity to the bile duct wall.

In this paper, we combined LSS and DRS modalities, designing and constructing a miniature LSS-DRS fiber optic probe compatible with the 1.2 mm diameter working channel of clinical cholangioscopes and ERCP catheters. This allowed us to investigate the internal cellular composition of the bile duct and detect malignant transformation (see Fig. 1). The technique was employed in vivo in 29 patients undergoing routine ERCP procedures.

## Results

### Extracting tissue structural and biochemical information with DRS and LSS

Diffuse reflectance spectroscopy (DRS) is a simple and convenient technique for non-invasive quantification of tissue optical properties without the use of contrast dyes, and with a high degree of biochemical sensitivity^22^. In DRS, the tissue surface is illuminated with broadband light, which undergoes multiple elastic scattering events and absorption within the tissue. When the non-absorbed portion of light eventually returns to the tissue surface, it carries quantitative information about the tissue, such as its structure and composition. This information can then be extracted from the measured reflectance using a steady-state spatially resolved diffusion theory model^34^. Unfortunately, the diffusion theory model is not accurate for small source-detector distances in the case of anisotropic scattering, which is typical for biological tissue. However, our group developed a phase function corrected diffusion theory^35^ that removes this deficiency, extending DRS to the small source-detector separation regime. For example, using results presented in Fig. 2a of ref 35. and for typical optical properties of tissue, such as Henyey–Greenstein phase function, highly forward peaked scattering with anisotropy factor *g* = 0.95, low absorption *μ*_a_ ≪ *μ'*_s_ (where *μ'*_s_ = (1 − *g*) *μ*_s_ is the reduced scattering coefficient and *μ*_a_ is the absorption coefficient), and small source-detector separations *ρµ'*_s_ < 1, the spatially dependent multiple scattering reflectance is simply *R*^ms^(*ρ*,*λ*) ≈ 0.4*R*^df^(*ρ*,*λ*), where *R*^df^(*ρ*,*λ*) is the spatially dependent diffuse reflectance derived in ref 34. This could be intuitively understood as highly forward peaked scattering makes it more difficult for the photons to turn around and therefore actual reflectance is redistributed compared to diffusion theory reflectance. In other words, many photons escaping the surface would travel from the area near the source and exit further away. Therefore, for the case of small source-detector separations

1 $${R}^{{ms}}\left(\rho,\,\lambda \right)\, \approx \, G\left(\lambda \right)\frac{{{\exp }}\left(-\sqrt{3}/2{\mu }_{a}{\mu }_{t}^{{\prime} }{\rho }^{2}\right)}{{\left(1+{{\mu }_{t}^{{\prime} }}^{2}{\rho }^{2}\right)}^{3/2}}$$

where *μ'*_t_ = *μ*_a_ + *μ'*_s_ is reduced transport coefficient, and the function

2 $$G\left(\lambda \right)\, \approx \, \frac{{\mu }_{s}^{{\prime} }{\mu }_{t}^{{\prime} }}{10\pi }{{\exp }}\left(-\sqrt{\frac{3{\mu }_{a}}{{\mu }_{t}^{{\prime} }}}\right)$$

is independent of the source-detector separation part of the reflectance.

**Fig. 2:**
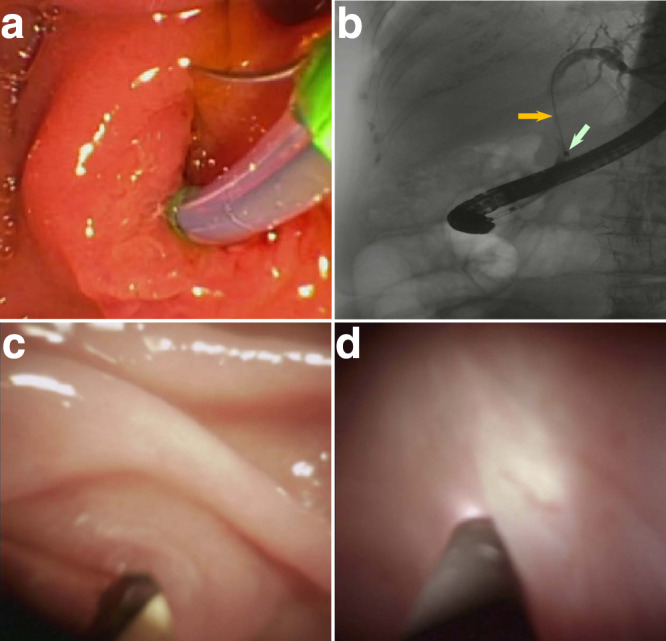
Visualization of in vivo LSS-DRS measurements in the bile duct with a cholangioscope or ERCP catheter. **a** Wire-guided biliary cannulation visualized using a duodenoscope. **b** X-ray image of the duodenoscope passing the ERCP catheter with LSS-DRS fiber probe into the pancreaticobiliary system, with the ERCP guidewire (orange arrow) extended to the intrahepatic duct. The radiopaque band at the distal end of the ERCP catheter, marked with a white arrow, provides a visual reference for positioning the LSS-DRS fiber probe. **c** Cholangioscope entering the pancreaticobiliary system via ampulla of Vater visualized with the duodenoscope. **d** LSS-DRS fiber probe, visualized using the cholangioscope, illuminates a location of the internal bile duct epithelial lining and the spectra is acquired.

It is important to emphasize here that the reflectance component of the signal given by (1) describes multiple scattering and absorption events in the deeper regions of the underlying connective tissue, but does not describe single backscattering events that take place in the uppermost cellular layer called the epithelium, where malignancy starts. Nevertheless, angiogenesis related changes in hemoglobin concentration and oxygen saturation of bile duct underlying connective tissue, along with potential structural changes, could be important indicators of malignancy and are worth considering. This can be achieved by extracting both structural and biochemical information from the reflectance component given in (1), which requires phenotypic properties of the bile duct mucosa, such as total hemoglobin concentration, hemoglobin oxygen saturation, and effective characteristic of the scatterers, often described by the scattering power, to be associated with wavelength dependent absorption and reduced scattering coefficients (see Methods).

As explained above, the multiple scattering component of the signal *R*^ms^(*ρ*,*λ*) does not describe the entire reflectance signal. To describe the entire signal, we need to take into account the single backscattering events in the epithelium, which is the superficial layer of mucosal tissue where malignancy starts. The technique that can analyze this component is light scattering spectroscopy, or LSS. The single backscattering component of the signal carries information about the malignant potential of epithelium, which can be evaluated using the so-called diagnostic parameter Δ introduced previously for Barrett’s esophagus^31^ and pancreatic cyst^33^ studies (see Methods).

To summarize, a DRS technique could be used to reconstruct several diagnostically important characteristics of the bile duct mucosa, such as hemoglobin concentration *c*_Hb_, hemoglobin oxygen saturation *α*, and scattering power *γ*, while a LSS technique could be used to calculate the diagnostic parameter Δ for bile duct epithelium.

### In vivo clinical LSS-DRS measurements in the bile duct

In vivo clinical LSS-DRS measurements were performed during ERCP procedures in 29 subjects. During the procedures, the patients were positioned on an X-ray table, lying on their side. An endoscope was inserted into the duodenum and a video-feed is transmitted to a screen located above the X-ray table. To allow easier examination, the stomach and duodenum were inflated by pumping air through the endoscope. When the duodenal papilla was located, a sphincterotome, which is a curved catheter with a cutting wire on its top, was inserted through the working channel of the endoscope and guided through the papillary opening (Fig. 2a). When the catheter was located inside the papilla, a dye was injected into the ducts, which provided X-ray contrast for the ductal tree (Fig. 2b). X-ray images could be obtained to observe the ducts and check for any abnormal narrowing or blockages. The cholangioscope or ERCP catheter was then placed inside the duodenoscope working channel and passed through the ampulla into the pancreaticobiliary system (Fig. 2c), providing access for the fiber probe (Fig. 1 and Supplementary Movie 1). After completing the visual examination of the bile duct, when the cholangioscope was employed, the LSS-DRS fiber optic probe (see Methods) was introduced through the cholangioscope accessory channel, or through the ERCP catheter, and extended into the bile duct. Spectra were acquired automatically when the LSS-DRS fiber probe was in close proximity and at approximately a right angle to the bile duct wall (Fig. 2d). We should note here that the diagnostic parameter Δ is not particularly sensitive to the angle to the bile duct wall (see Supplementary Information, Fig. S1). The fluoroscopy images and cholangioscope videos were recorded at each data collection site for site identification. A single spectroscopic data collection takes less than one second and the total spectroscopic measurement time is under 2 min, which includes probe insertion, collection of spectra at 2 to 14 sites, and probe removal. Small forceps biopsy specimens were collected for pathological examination from some of the sites according to the standard of care. The longitudinal spread of bile duct carcinoma is at least several millimeters^36^, well within the endoscopist’s capability to locate the same site using visual cues.

Overall, three to five spectra were obtained from each biopsy site before biopsy collection. After that, three to six biopsies per subject were collected from different locations of the bile duct. Three to ten spectra were obtained in the subjects where only brush cytology was collected. The approximate size of the spectroscopic collection site, which is 100 μm to 200 μm, was similar to the microscopic field of view employed for reaching the pathology based diagnosis. No adverse events were noted.

### Differentiating bile duct lesions in vivo with DRS and LSS

**Fig. 3:**
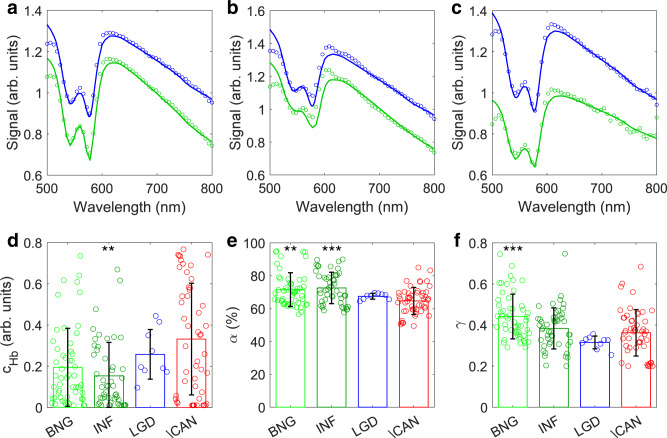
In vivo spectra collected during routine ERCP clinical procedures in bile ducts and tissue physiological parameters extracted from DRS. Typical white light spectra of benign (**a**), inflammation (**b**), and high-grade dysplasia (**c**) sites collected with LSS-DRS probe (circles) at 120 µm (blue) and 240 µm (green) source-detector separations and three-parameter fits (solid lines). Hemoglobin concentration *c*_Hb_ (**d**), oxygen saturation *α* (**e**), and scattering power *γ* (**f**) in the underlying connective tissue reconstructed from in vivo DRS data for all bile duct sites. The bar heights represent the mean values for each category and the error bars represent standard deviation. Each circle represents an individual measurement site, including 53 benign (BNG), 49 inflammation (INF), 9 low-grade dysplasia (LGD), and 50 high-grade dysplasia (HGD) or cancer (CAN) sites. A one-way Kruskal–Wallis test followed by Dunnett’s multiple comparisons test was used in (d) and (e), and a one-way ANOVA followed by Dunnett’s multiple comparisons test was used in (f) (**P = 0.004 for INF vs. HGD/CAN for hemoglobin concentration, **P = 0.003 for BNG vs. HGD/CAN and ***P = 0.0003 for INF vs. HGD/CAN for oxygen saturation, ***P = 0.0005 for BNG vs. HGD for scattering power).

Typical spectra collected in vivo with the LSS-DRS probe in high-grade dysplasia, inflammation, and benign bile duct sites are shown in Fig. 3a–c. The DRS model fits for the 120 µm and 240 µm source-detector distance fibers, also shown here, are excellent.

When the inverse DRS algorithm is applied to the in vivo reflectance spectra in the 500 nm to 800 nm wavelength range, it provides values for the hemoglobin concentration, oxygen saturation, and scattering power (see Fig. 3d–f) for the underlying connective tissue of each bile duct site, which includes 53 benign sites (BNG), 49 sites with inflammation (INF), 9 low-grade dysplasia sites (LGD), and 50 high-grade dysplasia (HGD) or early cancer sites (CAN). When the benign and inflammation sites are compared with the high-grade dysplasia and cancer sites, the mean hemoglobin concentration increases in the latter group, while both the mean hemoglobin oxygen saturation and scattering power decrease. Unfortunately, none of those changes are statistically significant and therefore could not be used for reliable differentiation of malignancy. This could be expected given that reflectance is dominated by the scattering in underlying connective tissue and only minimal changes can be observed there in early cancer and pre-cancer.

**Fig. 4:**
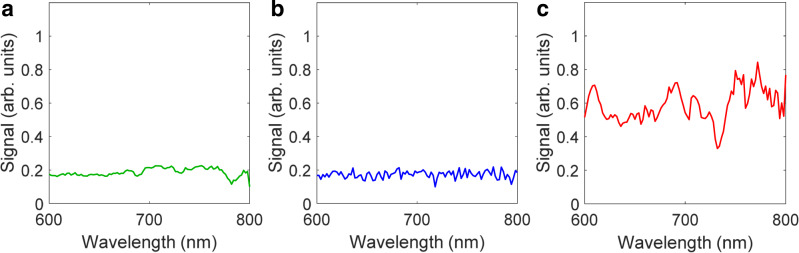
Single backscattering spectra. Typical single backscattering components reconstructed from the spatially gated white light data collected with the LSS-DRS probe in (**a**) benign, (**b**) inflammation, and (**c**) high-grade dysplasia bile duct sites.

The single backscattering component, on the other hand, depends mainly on scattering in the epithelium. It can be reconstructed from the spatially gated reflectance data in the 600 nm to 800 nm wavelength range, where tissue absorption can be ignored, by employing the procedure described in the Methods section, with the reconstructed single backscattering components for the typical high-grade dysplasia, inflammation, and benign bile duct sites shown in Fig. 4a–c. While a single backscattering spectrum of the high-grade dysplasia site exhibits a clear pattern, spectra for the inflammation and benign sites have significantly less structure. These differences are reflected in the diagnostic parameters Δ (see Method section for details).

The results of the study are summarized in Table 1. Biological tumor markers and imaging investigation in biliary structures have limited sensitivity and specificity, and as a result, definitive malignancy diagnosis can only be achieved with histopathology of biopsy samples or cytological evaluation of bile duct brushing. Within the patient set of 29 subjects, 13 had definitive histopathology diagnoses and 6 had definitive cytology diagnoses. All these cases involved consensus diagnosis by at least two staff pathologists. One more subject has survived for more than a year, with follow-ups showing no evidence of malignancy. Since untreated bile duct malignancy has a very poor prognosis with median survival reported to be <1 year^37–39^, survival for over a year after measurements can also be considered a reliable indicator of a non-malignant bile duct^40^. We consider the diagnosis of these 20 patients as reliable. Nine remaining patients have just recently been measured by our technique, and therefore do not have yet a sufficient survival follow-up after the measurement. For those subjects, an independent assessment by two expert gastroenterologists was obtained, who took into account the clinical history and ERCP imaging results, but were blinded to the LSS findings. The agreed consensus assessment was used as a secondary endpoint.

**Table 1:** In vivo differentiation of bile duct neoplasms in 29 subjects

Sub	Endoscopy modality	Histopathology	Cytology	Endoscopy assessment	Liver disease	Source of diagnosis	Diagnosis	Follow up	LSS (Δ)	LSS diagnosis
1	Cholangioscopy	HGAC	PMC, AC	Necrosis	No	Histopathology	HGAC	DBC	0.32	HGD/CAN
2	Cholangioscopy	Benign w/INF	–	Ulcerated	No	Histopathology	Benign	DUC	0.15	Benign
3	Cholangioscopy	Benign w/INF	NMC	Normal	No	Histopathology	Benign	Alive	0.14	Benign
4	Cholangioscopy	Benign w/INF	NMC	Normal	No	Histopathology	Benign	Alive	0.17	Benign
5	Cholangioscopy	–	–	Stone	No	GCA	Benign	Alive	0.17	Benign
6	Cholangioscopy	Hamartoma	−	–	No	Histopathology	Benign	Alive	0.03	Benign
7	Cholangioscopy	–	NMC	Normal	No	Cytology	Benign	DOA	0.05	Benign
8	Cholangioscopy	–	NMC	Normal	HS	Cytology	Benign	Alive	0.05	Benign
9	Cholangioscopy	Benign w/INF	–	Stone	LC, BRIC	Histopathology	Benign	Alive	0.07	Benign
10	Cholangioscopy	Benign w/INF	Atp	PSC	PSC	Histopathology	Benign	Alive	0.06	Benign
11	ERCP	LGD	–	Normal	No	Histopathology	LGD	ERCP (P)	0.15	Benign
12	Cholangioscopy	HGD	AGEC	MAS	No	Histopathology	HGD	CM (IP)	0.54	HGD/CAN
13	Cholangioscopy	–	–	Stone	PSC, HS	GCA	Benign	Alive	0.03	Benign
14	ERCP	HGAC	Atp	Stricture	LL	Histopathology	HGAC	CTX (IP)	0.40	HGD/CAN
15	ERCP	INF	Atp	Stricture	No	GCA	Benign	Alive	0.19	Benign
16	ERCP	–	–	Stone	No	GCA	Benign	Alive	0.07	Benign
17	ERCP	AC, LVI	–	Normal	No	Histopathology	AC, LVI	CTX (IP)	0.59	HGD/CAN
18	Cholangioscopy	–	–	Normal	No	GCA	Benign	Alive	0.07	Benign
19	ERCP	–	–	Normal	No	GCA	Benign	Alive	0.12	Benign
20	ERCP	PDAC	PMC	MAS	No	Cytology	PMC	CTA	0.59	HGD/CAN
21	Cholangioscopy	LGD	PMC, AC	Dilated	PSC, HC	Cytology	PMC, AC	CM (IP)	0.34	HGD/CAN
22	ERCP	–	–	Dilated	LL	GCA	Benign	Alive	0.17	Benign
23	ERCP	DA w/INF	NMC, Atp	Dilated	No	Cytology	Benign	Alive	0.14	Benign
24	Cholangioscopy	INF	–	Dilated	PHM	GCA	Benign	Alive	0.16	Benign
25	ERCP	–	–	Stone	No	GCA	Benign	Alive	0.12	Benign
26	ERCP	Benign w/INF	NMC	Dilated	LC	Histopathology	Benign	Alive	0.03	Benign
27	ERCP	AC	PMC, AC	Stone	No	Histopathology	PMC, AC	CTA	0.76	HGD/CAN
28	Cholangioscopy	–	–	Normal	No	GCA	Benign	Alive	0.04	Benign
29	ERCP	–	PMC, AC	MAS	No	Cytology	PMC, AC	CTX (IP)	0.18	Benign

*Sub* subject, *AC* adenocarcinoma, *HGAC* high-grade adenocarcinoma, *HGD* high-grade dysplasia, *LGD* low-grade dysplasia, *LVI* lymphovascular invasion, *PDAC* pancreatic ductal adenocarcinoma, *MAS* malignant appearing stricture, *PMC* positive for malignant cells, *NMC* negative for malignant cells, *Atp* atypical, *PSC* primary sclerosing cholangitis, *DA* duodenal adenoma, *AGEC* atypical glandular epithelial cells, *LC* liver cirrhosis, *BRIC* benign recurrent intrahepatic cholestasis, *LL* liver lesions, *INF* inflammation, *HS* hepatic steatosis, *HC* hepatitis C, *PHM* porta hepatis mass, *GCA* gastroenterologists’ consensus assessment, *CTX* chemotherapy, *CM* conservative management, *CTA* cancer treatment assessment, *DBC* die due to bile duct cancer, *DUC* die from unrelated causes, *DOA* die of old age, *P* planned, *IP* in progress.

Since all existing modalities for bile duct cancer treatment involve the entire bile duct and are not performed locally, it would make sense to characterize bile ducts with a single diagnostic parameter Δ, defined as the highest value of Δ obtained within that duct, to detect the presence of either high-grade dysplasia or early cancer in a particular patient. Furthermore, since bile duct and pancreatic cysts are characterized by a very similar type of lining, we decided to adopt the high-grade dysplasia and cancer diagnostic cutoff of 0.2 used in earlier pancreatic cyst studies^33^, instead of establishing it with a training cohort.

**Fig. 5:**
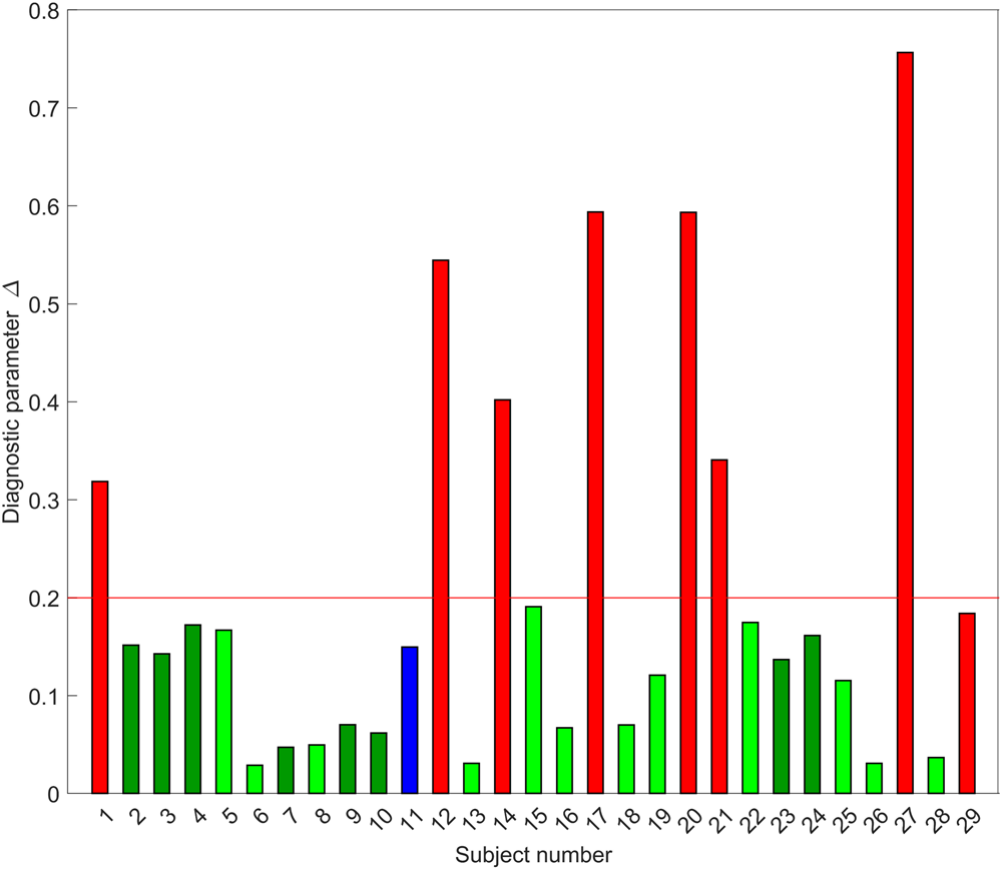
In vivo light scattering spectroscopic differentiation of bile duct malignancy in 29 subjects. The color of the bars represents the clinical diagnosis. Light green subjects with benign bile duct, dark green—benign with inflammation, blue—low-grade dysplasia, and red—high-grade dysplasia or cancer. The bar heights represent the light scattering spectroscopy based diagnostic parameter. The red line at 0.2 represents the high-grade dysplasia and cancer diagnostic cutoff established earlier in pancreatic cysts^33^.

The comparison of the diagnostic parameter Δ and the clinical diagnosis for each of the 29 patients is presented in Fig. 5. It appears to be significantly higher for the malignant bile ducts compared to the benign bile ducts with and without inflammation (*P* < 0.0001). In addition, the diagnostic parameter for the case of low-grade dysplasia, a category which requires observation with follow-ups but no intervention, was also significantly lower than that for the malignant cases. We note that in the presented study, both the clinicians and the investigators were double blinded during the measurements and outcome assessment. Our results show that all bile ducts with high-grade dysplasia and cancer, except one, were identified correctly by LSS. The only misidentified case had a Δ within 10% of the high-grade dysplasia and cancer diagnostic cutoff. The overall sensitivity for all 29 patients was 88% (95% CI: 53–99%) and specificity was 100% (95% CI: 85–100%).

## Discussion

Bile duct cancer is considered to be an incurable cancer that can rapidly become lethal, unless it can be detected early enough to be fully resected. Unfortunately, in most cases, it is detected at the advanced stages, which are inoperable at the time of diagnosis. Moreover, despite recent improvements in cross-sectional imaging and endoscopic technologies, differentiation of benign and malignant bile duct strictures remains a diagnostic challenge. Cross-sectional imaging lacks sensitivity to cellular structure and biochemical properties of the bile duct wall. While a combination of ERCP-based brush cytology and trans-papillary endobiliary forceps biopsy, which is often considered the gold standard, has a very high specificity of 97% to 100%, it offers sensitivity of only 47% to 86%^41^. Another very significant challenge with the current standard of care approaches is difficulty in acquiring tissue samples adequate for the histopathologic examination^42^.

**Fig. 6:**
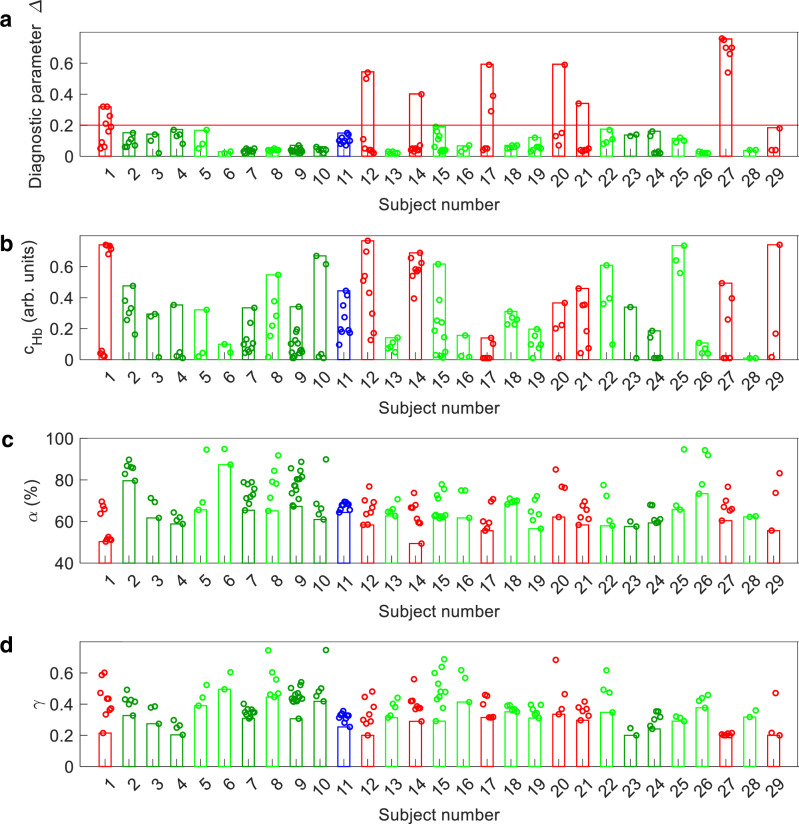
Tissue parameters reconstructed from in vivo spectroscopic measurements in the bile duct. The bar color represents clinical diagnosis. The red bars represent high-grade dysplasia or cancer, blue – low-grade dysplasia, green—benign with inflammation and bright green—benign. Bar heights represent tissue parameters reconstructed from in vivo spectroscopic measurements with the highest LSS-based diagnostic parameter ∆ (**a**), hemoglobin concentration *c*_Hb_ (**b**), lowest oxygen saturation *α* (**c**), and DRS-based scattering power *γ* (**d**) among all measurement sites of each subject. The red line at 0.2 in (**a**) represents the high-grade dysplasia and cancer diagnostic cutoff established earlier in pancreatic cysts^33^.

Here, we report an endoscopic optical light scattering technique for clinical evaluation of the malignant potential of the bile duct. This technique employs an ultraminiature spatial gating fiber optic probe compatible with clinical cholangioscopes and ERCP catheters. The spectra are acquired automatically when the probe is in close proximity and at approximately a right angle to the bile duct wall. Therefore, a visual examination of the bile duct wall with a cholangioscope is useful but not required. The technique allows investigation of the internal cellular composition of the bile duct epithelium with LSS, and phenotypic properties of the underlying connective tissue hemoglobin concentration and hemoglobin oxygen saturation with diffuse reflectance spectroscopy. In the in vivo double-blind prospective pilot study described here involving 29 patients undergoing routine ERCP procedures, the technique detected biliary duct pre-cancer and early cancer with a 97% accuracy, with a 95% confidence interval (CI) of 83-100%. This diagnostic accuracy was achieved by employing the LSS information alone. The study was performed in a perspective way and employed the predetermined diagnostic cutoff for the LSS diagnostic parameter Δ, established previously in pancreatic cyst studies^33^. The use of the same diagnostic cutoff can be justified by taking into account similarities in the epithelial lining of the bile duct and mucinous pancreatic cysts. Malignancy classification based only on the LSS diagnostic parameter (Fig. 6a) gives a sensitivity of 88% (95% CI: 53–99%) and a specificity of 100% (95% CI: 85–100%), a significant improvement over the standard of care ERCP-based diagnosis. This could be expected given that the LSS signal is dominated by the scattering in the epithelial lining where the majority of the malignant transformations take place.

Epithelial cells found in the bile duct might have various shapes. Intrahepatic biliary epithelium is lined by cuboidal cells, extrahepatic biliary epithelium is lined by columnar cells, and hilar epithelium is lined by both cuboidal and columnar cells. However, despite the shape differences, high-grade dysplasia develops in all these epithelial cells with similar important histopathological characteristics: nuclear enlargement, hyperchromasia, and cell crowding^43–45^. The same is true for pancreratic^46^, ampullary^47^, and gall bladder^48^ malignancies, which can also be present within the biliary tree in columnar and cuboidal epithelial cells. One of the main reasons LSS detects high-grade dysplasia and cancer in these diverse cell types is its inherent sensitivity to each of the histopathological characteristics listed above.

Out of 29 consecutively enrolled patients, 17 were without any suspicion for cholangiocarcinoma. For this subgroup, the diagnostic accuracy is 100% (95% CI: 82-100%), very similar to the diagnostic accuracy of the entire pool of 29 subjects. Interestingly, among this subgroup of patients without any suspicion for cholangiocarcinoma, 3 subjects were later diagnosed by histopathology or cytology to have cholangiocarcinoma and 1 to have pancreatic ductal adenocarcinoma of the bile duct. All 4 patients were correctly categorized by our technology prior to the diagnosis.

At the same time, classification capabilities of the hemoglobin concentration, hemoglobin oxygen saturation and subepithelial scattering power, which can be reconstructed from the DRS data (Fig. 6b–d), remains an open question. The wall of the bile duct below a relatively thin epithelial lining is a mixture of muscle and dense tissue fibers called the fibromuscular layer^49^. Neither of these change significantly during early stages of malignant transformations, and thus, the subepithelial scattering power reconstructed from the DRS data should not be too sensitive to malignancy. While angiogenesis, the formation of new blood vessels which provide nutrition to malignant cells, is considered to be an important phenomenon in neoplasia development, the related increase in hemoglobin concentration is characteristic of the later stages of malignancy. The same is true for oxygen saturation. As a tumor grows, oxygen is rapidly consumed by the proliferating tumor cells, leading to a hypoxic microenvironment^50,51^ but once again this phenomenon is usually observed during later stages of tumorigenesis.

Though the increase in the hemoglobin concentration could be explained by angiogenesis and the decrease in oxygen saturation from ~72% on average for benign and inflammation sites to below 65% on average for high-grade dysplasia and cancer sites is consistent with the changes that has previously been observed in various gastrointestinal organs such as esophagus^52^, stomach^53^, and colon^22^, none of these changes could be used for reliable differentiation of malignancy.

To test the classification capabilities of DRS-based parameters, we employed the receiver operating characteristic (ROC) approach to evaluate their potential sensitivities and specificities (see Fig. S2). Since the LSS-based classification is capable of a 100% specificity and the standard of care ERCP-based diagnosis, while not particularly sensitive, also provides close to a 100% specificity^41^, it would make sense to compare sensitivities of the DRS-based diagnosis for the same specificity conditions. Using ROC curves at a 100% specificity, we observed a 38% sensitivity for classification using hemoglobin concentration, a 50% sensitivity for hemoglobin oxygen saturation, and a 25% sensitivity for the underlying connective tissue scattering power. However, it is important to note that unlike the classification based on the predetermined LSS diagnostic parameter, the DRS-based classification is not performed in a perspective manner. Nevertheless, adding DRS-based parameters to the LSS-based classification could in principle further improve the already excellent sensitivity of the LSS-based classification, and additional studies are necessary to check this.

Currently, surgery for resectable bile duct cancers require not only removal of the bile duct, but also removal of part of the liver, nearby lymph nodes, and the gallbladder. In many cases, this is not enough and the Whipple procedure is performed. During the Whipple procedure, which has high morbidity and mortality rates^54^, part of the pancreas and small intestine are additionally removed. The major reason that these surgeries are so extensive and dangerous is because tumors are found very late. A technique capable of reliably detecting pre-cancer or early cancer of the bile duct can lead not only to improving the prognosis for bile duct cancer patients, but could also lead to a different type of treatment, where only a small part of the bile duct is either removed or even radiofrequency ablated. This is especially true if that technique could specify the location of pre-cancer or early cancer.

The review of the LSS data shows that in one of the subjects (subject 17, Fig. 6a and Table 1) there are several locations in the extrahepatic duct which have diagnostic parameter Δ above the malignancy diagnostic cutoff, while all the intrahepatic duct locations are classified as benign. At the same time, two subjects (subjects 14 and 21, Fig. 6a and Table 1) have single LSS detected malignant sites in the intrahepatic duct and no malignancy anywhere else. These LSS findings are consistent with earlier histopathology observations that malignant transformations which arise in the region of the duodenal papilla are extended only to the extrahepatic bile duct and do not extend to the intrahepatic bile duct^55^, a fact which was previously associated with the presence of field carcinogenesis^56^. It was recently demonstrated that field cancerization changes could be detected in ex vivo bile duct specimens using spatial-domain low-coherence quantitative phase microscopy (SL-QPM)^57^, a technique which targets spatial fluctuations of the refractive index as a marker of malignancy. LSS, on the other hand, targets refractive index-related spectral fluctuations, which might therefore have a similar nature.

In conclusion, we present here a minimally invasive optical spectroscopic technique showing that it is possible to reliably identify biliary duct pre-cancer and early cancer in vivo non-invasively. A major advantage of the technique is that the developed relatively inexpensive spatial gating fiber optic probe could be easily miniaturized to fit the most restricted spaces of cholangioscopes and ERCP catheters. About half a million ERCP procedures with conventional ERCP catheters are performed annually in the United States^58^. The technique presented here works with both conventional ERCP catheters and cholangioscopes. In this study, 13 procedures were performed with conventional ERCP catheters, while 16 procedures were performed with cholangioscopes. It was the clinician’s call as to which of the two methods was used, and this decision was not influenced by the study requirements. Also, the choice did not affect the LSS-DRS measurements in terms of procedure, or in terms of the spectroscopic measurement outcomes. Importantly, no special training is needed for clinicians to operate the system, as it collects data automatically and positioning of the fiber optic probe is very similar to positioning biopsy forceps. In a pilot in vivo study of 29 patients undergoing routine ERCP procedures, the technique detected morphological changes in the epithelium, reliably identifying biliary duct pre-cancer and cancer. The optical measurements are rapid and performed in <2 min. Therefore, the presented technique offers great promise for the early detection of dysplasia in the bile duct.

## Methods

This research complies with all relevant ethical regulations and was reviewed and approved by the Institutional Review Board (IRB protocol #: 2004P000198) at Beth Israel Deaconess Medical Center (BIDMC). Twenty-nine consecutively enrolled subjects, of which 15 were females and 14 were males, gave signed informed consent approved by the BIDMC IRB. The median age of subjects was 70 years (range 49–90 years). No compensation was provided for participating in the study.

### Absorption and reduced scattering coefficients of mucosa

For mucosal tissue, hemoglobin (Hb) is the only notable light absorber in the visible spectrum. The total absorption coefficient of hemoglobin in the subepithelial underlying connective tissue *μ*_a_(*λ*) can be presented as a weighted sum of the wavelength dependent extinction coefficients for oxygenated and deoxygenated forms of hemoglobin

3 $${\mu }_{a}\left(\lambda \right)={c}_{{Hb}}\left[\alpha \,\cdot \,{\varepsilon }_{{Hb}{O}_{2}}\left(\lambda \right)+\left(1-\alpha \right){\varepsilon }_{{Hb}}\left(\lambda \right)\right]$$

where *c*_*Hb*_ is the total hemoglobin concentration, *α* is the hemoglobin oxygen saturation parameter, and ${\varepsilon }_{{Hb}}\left(\lambda \right)$ and ${\varepsilon }_{{Hb}{O}_{2}}\left(\lambda \right)$ are the well documented^59^ wavelength dependent extinction coefficients of deoxygenated and oxygenated hemoglobin, respectively. We should note here that significant absorption peaks for oxygenated and deoxygenated hemoglobin located in the so-called Soret band^60^ between 400 nm and 450 nm saturate the absorption to such an extent that the inverse DRS algorithm becomes insensitive to the more important 500 nm to 600 nm wavelength region. This then reduces the accuracy of the algorithm when extracting tissue oxygenation. In addition, the 400 nm to 500 nm wavelength region also features interference from bilirubin absorption^61,62^. Therefore, DRS data analysis in this study is performed in the 500 nm to 800 nm wavelength region.

The reduced scattering coefficient of the subepithelial underlying connective tissue scatterers *μ'*_s_(*λ*) can be approximated as

4 $${\mu }_{s}^{{\prime} }\left(\lambda \right)={\rho }_{s}{\sigma }_{s}\left(\lambda \right)$$

where the scattering properties of the underlying connective tissue are associated with a single type of effective scatterers with density *ρ*_s_ and reduced scattering cross section *σ*_s_(*λ*). The reduced scattering cross section, *σ*_s_(*λ*), depends on the size and shape of the effective scatterers, along with the refractive indices of the scatterers and the surrounding medium. One can assume the average scatterers in the underlying connective tissue to be spherical particles of a certain diameter and use Mie scattering theory to evaluate *σ*_s_(*λ*)^22^, further approximating the reduced scattering coefficient as^63,64^

5 $${\mu }_{s}^{{\prime} }\left(\lambda \right)={\rho }_{s}{\sigma }_{s}\left({\lambda }_{0}\right){\left({\lambda }_{0}/\lambda \right)}^{\gamma }$$

where *λ*_0_ is an arbitrary wavelength and *γ* is the scattering power.

### Reconstruction of single backscattering component

**Fig. 7:**
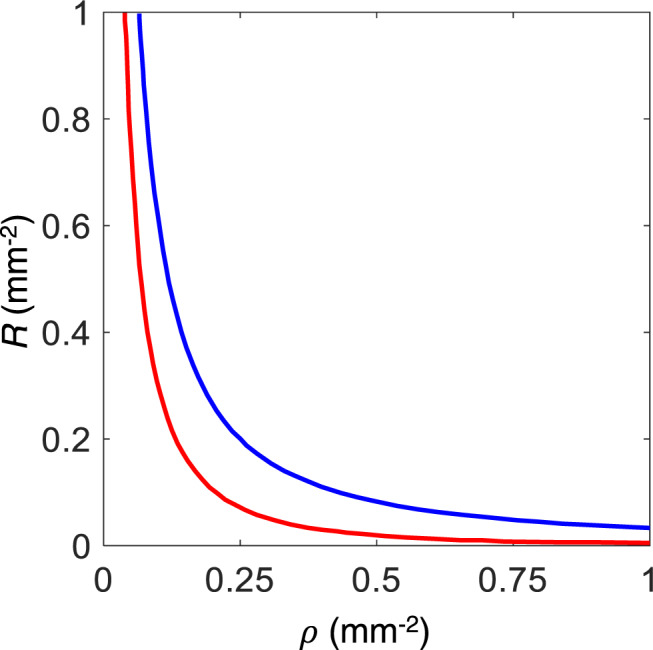
Comparison of the contributions of the multiple scattering and single backscattering components. Reflectance (*R*) is plotted vs. source-detector separation (*ρ*). The calculations are performed for typical optical properties of tissue: Henyey–Greenstein phase function, *g* = 0.9, *µ'*_s_ = 1.0 mm^−1^, and *µ*_a_/*µ'*_s_ = 0.01. Red line—single backscattering component, blue line—multiple scattering components.

The single backscattering signal can be determined if the diffuse component is removed by one of the gating approaches, such as polarization gating^31,65^ or spatial gating^33^. Here we employed spatial gating, which uses the fact that the diffuse and single backscattering components depend very differently on the source-detector distance. The LSS-DRS fiber optic probe used in our studies provides three possible source-detector separations with the smallest being 120 µm and largest being 240 µm. An increase in the source-detector distance causes the single backscattering component to decrease significantly faster than the diffuse component^35^. This significant reduction means that the single backscattering component can be ignored for the 240 µm source-detector separation fiber (see Fig. 7). That gives us the following system of equations for the total reflectance signals collected by the fibers with *r*_1_ = 120 µm and *r*_2_ = 240 µm source-detector separations

6 $${R}^{{{{{{\rm{ex}}}}}}}\left({r}_{1},\, \lambda \right) 	={R}^{{{{{{\rm{ms}}}}}}}\left({r}_{1},\, \lambda \right)+{R}^{{{{{{\rm{sb}}}}}}}\left(\lambda \right)\\ {R}^{{{{{{\rm{ex}}}}}}}\left({r}_{2},\, \lambda \right) 	={R}^{{{{{{\rm{ms}}}}}}}\left({r}_{2},\, \lambda \right)$$

where *R*^ex^(*r, λ*) is the experimental spectrum collected by a collection fiber at distance r from the source fiber, *R*^ms^(*r, λ*) is multiple scattering component described by (1) and *R*^sb^(*λ*) is the single backscattering component.

We can see from (1) that for the small source-detector separations, the only notable difference in the shape of the wavelength dependent multiple scattering component of the signal *R*^ms^(*r, λ*) might be observed in the wavelength region where the contribution of the exponential term is significant, in other words, where there is significant hemoglobin absorption. Since hemoglobin absorption is rather weak in the wavelength range from 600 to 800 nm, the multiple scattering components are approximately proportional to each other here, with the constant proportionality coefficient *ξ* ≈ [(1 + *μ'*_s_(*λ*_0_)^2^*r*_1_^2^)/(1 + *μ'*_s_(*λ*_0_)^2^*r*_2_^2^)]^3/2^, where *λ*_0_ = 700 nm. The coefficient *ξ* could be further calibrated empirically to take into account the differences in the collection efficiency of the fibers and uncertainty in the reduced scattering coefficient value at *λ*_0_.

**Fig. 8:**
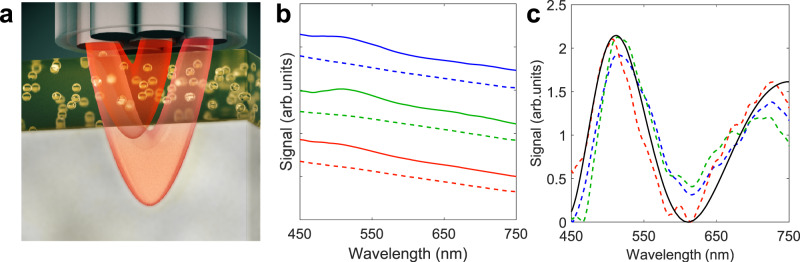
Two-layered tissue phantom experiments representing epithelium and underlying connective tissue. **a** Schematic of the two-layered tissue phantom experiment. **b** Total reflectance signals collected from two-layered phantoms at *r*_1_ = 120 µm (solid lines) and *r*_2_ = 240 µm (dashed lines) source-detector separations for reduced scattering coefficients of the bottom layer *µ'*_s_ = 1 mm^−1^ (red), 1.5 mm^−1^ (green) and 2 mm^−1^ (blue). **c** Reconstructed single backscattering components of the top layer for bottom layers with 1 mm^−1^ (red), 1.5 mm^−1^ (green) and 2 mm^−1^ (blue) reduced scattering coefficients and Mie theory calculations for 0.5 μm diameter polystyrene microspheres in backscattering direction (black line).

We should note here that to remove the optical contribution of the underlying connective tissue, we do not need to know the exact dependence of the multiple scattering component on the optical parameters of this layer, with knowledge of the empirical coefficient *ξ* being more than sufficient. To confirm this, we constructed three two-layered tissue phantoms with the top 200 μm thick layer of the phantom representing epithelium and consisting of 0.5 μm diameter polystyrene microspheres gelled in agarose (see Fig. 8a). The concentration of beads in the top layer is chosen so that the reduced scattering coefficient of the layer is *μ*'_s_ = 0.3 mm^−1^ for all three phantoms. The bottom 1.5 mm thick layer consisted of milk gelled in agarose and represented the underlying connective tissue. The concentration of milk gelled in agarose differed for the three phantoms (*μ*'_s_ = 1 mm^−1^, 1.5 mm^−1^ and 2 mm^−1^ at 630 nm) and was chosen so that the reduced scattering coefficient of the bottom layer covered the range of optical parameters of the underlying connective tissue. The absorption of water, gel, polystyrene beads and milk is insignificant, and no additional absorbers have been added. The measurements were performed in the 450–750 nm wavelength range.

The total reflectance spectra for each phantom at each source-detector separation are rather different (Fig. 8b). They also exhibit very little resemblance with the results from Mie scattering theory for the top layer. However, after the multiple scattering component is removed using the relationship

7 $${R}^{{{{{{\rm{sb}}}}}}}\left(\lambda \right)\,\approx \,{R}^{{{{{{\rm{ex}}}}}}}\left({r}_{1},\, \lambda \right)-\xi {R}^{{{{{{\rm{ex}}}}}}}\left({r}_{2},\, \lambda \right)$$

excellent agreement is achieved with Mie theory for all three phantoms, allowing the scatterers sizes in the top layer to be precisely reconstructed (Fig. 8c). The reconstructed size for the microspheres with nominal diameter of 500 nm was 486 nm, which is within the standard deviation of the microsphere sizes provided by the manufacturer.

In the case of tissue, the single backscattering component can be evaluated from (7) in the 600–800 nm wavelength range where there is no significant hemoglobin absorption. From here we can calculate the diagnostic parameter

8 $$\Delta=\frac{1}{2}\mathop{\sum}\limits_{\lambda }{\left({\bar{R}}^{{{{{{\rm{sb}}}}}}}\left(\lambda \right)-\left\langle {\bar{R}}^{{{{{{\rm{sb}}}}}}}\left(\lambda \right)\right\rangle \right)}^{2}$$

where ${\bar{R}}^{{{{{{\rm{sb}}}}}}}\left(\lambda \right)$ and $\langle {\bar{R}}^{{{{{{\rm{sb}}}}}}}\left(\lambda \right)\rangle$ are the normalized and root-mean-square–normalized single backscattering components, respectively, which are summed across all spectral points *λ*^31^.

### Clinical system

The clinical system employed a 75 W fiber-coupled xenon short arc light source (SLS205, Thorlabs) as a source of the white light and three spectrometers (AvaSpec-2048-USB2, Avantes) for the collection of spectra at three source-detector separations. The system was assembled on a cart for convenient transportation to the clinic (see Fig. 1a). The light was delivered and collected using a custom built miniature spatially gated LSS-DRS fiber optic probe. A custom graphical user interface (GUI) coded in MATLAB was used to control the system and collect data. The cost of the equipment was ~$18,000.

The probe consisted of seven fibers, each with a 100 μm core diameter and NA = 0.22. A single fiber located in the outer ring of the LSS-DRS probe served as the delivery fiber and three fibers positioned at 120 µm, 220 µm and 240 µm center-to-center from the delivery fiber served as the collection fibers. At the proximal end, each collection fiber was coupled to an individual spectrometer channel. In this study the closest (120 μm) and farthest (240 μm) fibers, providing two distinct source-detector separations, were employed for the data analysis. The overall diameter of the probe was 0.9 mm, making it compatible with the 1.2 mm working channel of all currently used cholangioscopy systems (Boston Scientific SpyGlass, Pentax FCP-9P and FCN−15X, Olympus CHF-BP30 and CHF-CB30L/S) and all ERCP catheters.

### Statistical analysis

Mean and standard deviation of the mean (SD) are used for bar graphs. Shapiro-Wilk normality tests were used before proceeding to any analysis. To compare HGD/CAN with LGD, INF, and BNG separately, a one-way ANOVA followed by Dunnett’s multiple comparisons test was used when data was normally distributed, while a one-way Kruskal–Wallis test followed by Dunnett’s multiple comparisons test was used when data was not normally distributed. To compare HGD/CAN with all other categories combined, a two-tailed Mann–Whitney test was used. All statistical analysis was performed using GraphPad Prism 8. *P*-values < 0.05 were considered statistically significant. The investigators were double blinded during the measurements and outcome assessment.

### Reporting summary

Further information on research design is available in the Nature Portfolio Reporting Summary linked to this article.

## Supplementary information

Supplementary Information

Description of Additional Supplementary Files

Supplementary Movie 1

Reporting Summary

## Data Availability

The datasets generated during the current study are available in the Figshare repository, at the link: 10.6084/m9.figshare.21552576.

## Code availability

The diagnostic algorithm is described in detail in the Methods section. The source code used in this work is available for research purposes from the corresponding authors upon reasonable request.
