# Supplementary material for: In vivo detection of bile duct pre-cancer with endoscopic light scattering spectroscopy
Source: Nat Commun. 2023 Jan 7;14:109. doi: 10.1038/s41467-022-35780-7 (PMC9825389; doi:10.1038/s41467-022-35780-7)
Supplement: Supplementary file 1 — Supplementary Information [file 41467_2022_35780_MOESM1_ESM.pdf]

Supplementary Information

In vivo detection of bile duct pre-cancer with endoscopic light scattering

Douglas K. Pleskow[1,2,4], Mandeep S. Sawhney[2,4], Paul K. Upputuri[1,4], Tyler M. Berzin[2,4], Mark F. Coughlan[1], Umar Khan[1], Maria Glyavina[1], Xuejun Zhang[1], Liming Chen[1], Conor J. Sheil[1], Jonah M. Cohen[2], Edward Vitkin[1], Yuri N. Zakharov[1], Irving Itzkan[1], Lei Zhang[1*], Le Qiu[1*] & Lev T. Perelman[1,3*]

[1]Center for Advanced Biomedical Imaging and Photonics
Division of Gastroenterology, Department of Medicine
Beth Israel Deaconess Medical Center
Harvard University

[2]Center for Advanced Endoscopy
Division of Gastroenterology, Department of Medicine
Beth Israel Deaconess Medical Center
Harvard University

[3]Biological and Biomedical Sciences Program
Harvard University

[4]These authors contributed equally: Douglas K. Pleskow, Mandeep S. Sawhney, Paul K. Upputuri, Tyler M. Berzin

[*]Email: lzhang11@bidmc.harvard.edu, lqiu@bidmc.harvard.edu; lperelman@fas.harvard.edu

Supplementary Figure 1

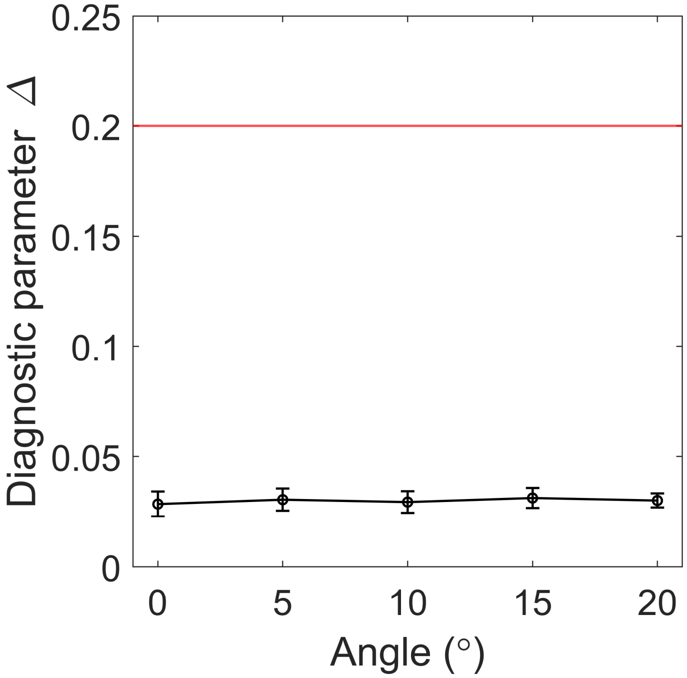

Fig. S1 Dependence of the diagnostic parameter on the orientation of the LSS-DRS fiber probe. Diagnostic parameter Δ obtained for a lip epithelium of a healthy volunteer (black circles) as a function of the angle of incidence. At each angle, data are presented as means ± SD of 10 measurements . The red line at 0.2 represents the high-grade dysplasia and cancer diagnostic cutoff, and is provided as a reference. The variability of the data is less than 3% of that cutoff.

Supplementary Figure 2

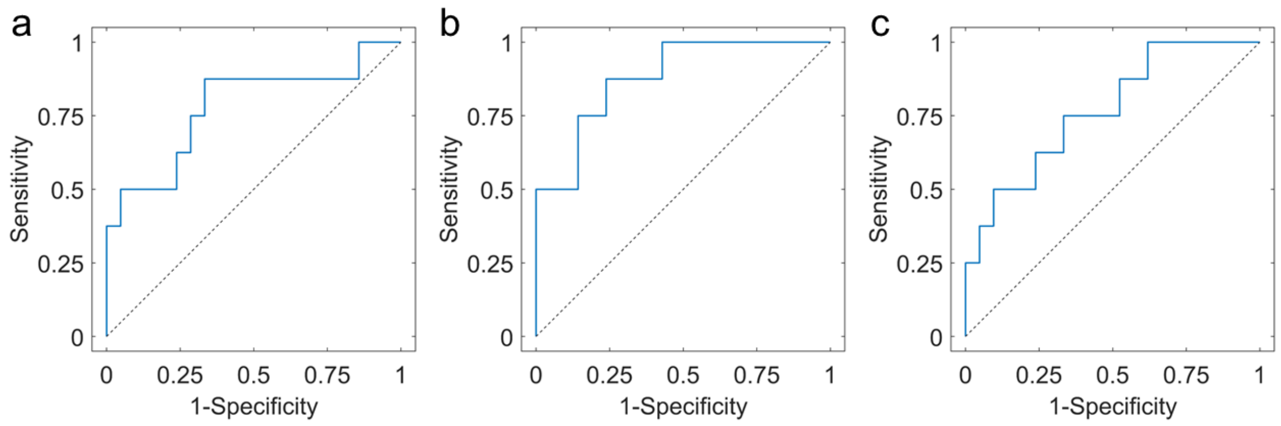

Fig. S2 Receiver operating characteristic (ROC) curves for *in vivo* DRS-based classification of malignancy in the bile duct. ROC curves of (**a**) hemoglobin concentration, (**b**) hemoglobin oxygen saturation, and (**c**) scattering power in the underlying connective tissue. The areas under the curves (AUCs) are 0.78, 0.88 and 0.77, respectively. The AUC for LSS-based diagnostic parameter (not shown) is 0.99.
