# Supplementary material for: In vivo detection of bile duct pre-cancer with endoscopic light scattering spectroscopy
Source: Nat Commun. 2023 Jan 7;14:109. doi: 10.1038/s41467-022-35780-7 (PMC9825389; doi:10.1038/s41467-022-35780-7)
Supplement: Supplementary file 2 — Description of Additional Supplementary Files [file 41467_2022_35780_MOESM2_ESM.pdf]

Description of Additional Supplementary Files

File Name: Supplementary Movie 1
Description: Animation of the in vivo clinical spectroscopic measurements in the bile duct.
