# Supplementary material for: In vivo detection of bile duct pre-cancer with endoscopic light scattering spectroscopy
Source: Nat Commun. 2023 Jan 7;14:109. doi: 10.1038/s41467-022-35780-7 (PMC9825389; doi:10.1038/s41467-022-35780-7)
Supplement: Supplementary file 4 — Reporting Summary [file 41467_2022_35780_MOESM4_ESM.pdf]

nature research

Corresponding author(s): Lei Zhang, Le Qiu & Lev T. Perelman

Last updated by author(s): Nov 17, 2022

Reporting Summary

Nature Research wishes to improve the reproducibility of the work that we publish. This form provides structure for consistency and transparency in reporting. For further information on Nature Research policies, see our Editorial Policies and the Editorial Policy Checklist.

Statistics

For all statistical analyses, confirm that the following items are present in the figure legend, table legend, main text, or Methods section.

n/a	Confirmed	
☐	☒	The exact sample size (*n*) for each experimental group/condition, given as a discrete number and unit of measurement
☐	☒	A statement on whether measurements were taken from distinct samples or whether the same sample was measured repeatedly
☐	☒	The statistical test(s) used AND whether they are one- or two-sided<br>*Only common tests should be described solely by name; describe more complex techniques in the Methods section.*
☐	☒	A description of all covariates tested
☐	☒	A description of any assumptions or corrections, such as tests of normality and adjustment for multiple comparisons
☐	☒	A full description of the statistical parameters including central tendency (e.g. means) or other basic estimates (e.g. regression coefficient) AND variation (e.g. standard deviation) or associated estimates of uncertainty (e.g. confidence intervals)
☐	☒	For null hypothesis testing, the test statistic (e.g. *F*, *t*, *r*) with confidence intervals, effect sizes, degrees of freedom and *P* value noted<br>*Give P values as exact values whenever suitable.*
☒	☐	For Bayesian analysis, information on the choice of priors and Markov chain Monte Carlo settings
☒	☐	For hierarchical and complex designs, identification of the appropriate level for tests and full reporting of outcomes
☒	☐	Estimates of effect sizes (e.g. Cohen's *d*, Pearson's *r*), indicating how they were calculated

Our web collection on statistics for biologists contains articles on many of the points above.

Software and code

Policy information about availability of computer code

Data collection	Data was collected during the clinical endoscopy procedures using the MATLAB-based acquisition software we developed.
Data analysis	MATLAB 2016b;<br>GraphPad Prism v8.

For manuscripts utilizing custom algorithms or software that are central to the research but not yet described in published literature, software must be made available to editors and reviewers. We strongly encourage code deposition in a community repository (e.g. GitHub). See the Nature Research guidelines for submitting code & software for further information.

Data

Policy information about availability of data

All manuscripts must include a data availability statement. This statement should provide the following information, where applicable:

- Accession codes, unique identifiers, or web links for publicly available datasets
- A list of figures that have associated raw data
- A description of any restrictions on data availability

The datasets generated during the current study are available in the Figshare repository, at the link: https://doi.org/ 10.6084/m9.figshare.21552576

Field-specific reporting

Please select the one below that is the best fit for your research. If you are not sure, read the appropriate sections before making your selection.

☒ Life sciences ☐ Behavioural & social sciences ☐ Ecological, evolutionary & environmental sciences

For a reference copy of the document with all sections, see nature.com/documents/nr-reporting-summary-flat.pdf

Life sciences study design

All studies must disclose on these points even when the disclosure is negative.

Sample size	The final sample size is sufficient to demonstrate that the endoscopic optical light scattering technique provides a statistically significant improvement in diagnostic accuracy compared to brush cytology and biopsy, which are the current standard of care diagnostic techniques.
Data exclusions	All patients that had been consented for data collection were included.
Replication	All measurements have been performed with appropriate replicates, as described in the Figure legends and Methods section. Data was collected in 8 subjects with malignant bile duct diagnoses and 21 subjects with benign bile duct diagnoses.
Randomization	Randomization is not relevant to our study, as this is not a case-control study.
Blinding	The investigators were double blinded during the measurements and outcome assessment.

Reporting for specific materials, systems and methods

We require information from authors about some types of materials, experimental systems and methods used in many studies. Here, indicate whether each material, system or method listed is relevant to your study. If you are not sure if a list item applies to your research, read the appropriate section before selecting a response.

Materials & experimental systems

n/a	Involved in the study	
☒	☐	Antibodies
☒	☐	Eukaryotic cell lines
☒	☐	Palaeontology and archaeology
☒	☐	Animals and other organisms
☐	☒	Human research participants
☒	☐	Clinical data
☒	☐	Dual use research of concern

Methods

n/a	Involved in the study	
☒	☐	ChIP-seq
☒	☐	Flow cytometry
☒	☐	MRI-based neuroimaging

Human research participants

Policy information about studies involving human research participants

Population characteristics	1.Male or female > 18 years of age<br>2.Willing and able to comply with Registry procedures and provide written informed consent to participate in the Registry<br>3.Indicated for ERCP
Recruitment	Measurements were performed in consecutive patients undergoing ERCP procedures at the BIDMC Center for Advanced Endoscopy to diagnose bile duct problems. We explained the procedure, indications, preparation, and potential complications to the subjects who indicated their understanding and signed the corresponding consent forms approved by the BIDMC Institutional Review Board (IRB). Subjects older than 18 years of age, of which 15 were female and 14 were male, were examined with either a cholangioscope or ERCP catheter. The median age of subjects was 70 years (range 49 to 90 years). No compensation was provided for participating in the study.
Ethics oversight	The BIDMC IRB approved the study protocol (IRB protocol #: 2004P000198).

Note that full information on the approval of the study protocol must also be provided in the manuscript.
